# Tweets Surrounding Pharmaceutical Drug Brands With Top Direct-to-Consumer TV-Advertising Budgets: Social Media Listening Study

**DOI:** 10.2196/85641

**Published:** 2026-06-18

**Authors:** Wendy Macias, María Belén Navarro Castillo

**Affiliations:** 1 Department of Strategic Communication, Texas Christian University, TCU Box 298065, Fort Worth, TX, 76129, United States, 1 817 257 4577; 2 AECOM, Dallas, TX, United States

**Keywords:** social media listening, direct-to-consumer pharmaceutical advertising, agenda setting, uses and gratifications, patient engagement

## Abstract

**Background:**

Direct-to-consumer (DTC) pharmaceutical advertising allocates billions annually in the United States; however, the analysis of conversations on social media about DTC drugs remains sparse. Twitter (subsequently rebranded X) is serving as a forum for pharmaceutical companies, their constituents, and social media health influencers to discuss topics related to DTC drugs with high advertising budgets.

**Objective:**

This study aims to examine user-generated topics discussed for the highest-budgeted DTC pharmaceutical drugs and to identify the agenda-setting themes and uses and gratifications that emerged.

**Methods:**

This social media listening study used Brandwatch to analyze Twitter conversations (August 2021-August 2023) surrounding top-budgeted DTC brands from 2020 to 2022. A dataset of 44,700 mentions from 26,800 unique authors was analyzed for content, sentiment, and thematic relevance using agenda-setting and uses and gratifications frameworks.

**Results:**

Four dominant themes emerged: (1) patient experiences/testimonials, (2) drug pricing/insurance concerns, (3) pharmaceutical news, and (4) advertising commentary. The content shows a range of potentially agenda-setting topics, including pharmaceutical companies, their DTC drugs, patient experiences, costs, insurance, and advertising commentary. The uses and gratifications we found included information, entertainment, economic benefits, and social benefits, with information being the most prevalent.

**Conclusions:**

Our study provides an important glimpse into what is being discussed on Twitter by, with, and about pharmaceutical drugs that spend billions on advertising each year [1]. Although more tweets were neutral than positive or negative, the overall sentiment of the top terms was negative. The predominance of negative sentiment in our findings suggests that many social media users express apprehensions or criticisms about these drugs, highlighting the need for improved communication, transparency, and engagement strategies. Further implications are also discussed surrounding public policy and industry practice. Suggested future research directions include NodeXL network analysis and qualitative inquiry into user motivations.

## Introduction

### Background

Around 60% of the world’s population use social media [1], and surveys have shown that up to 26% of Americans use social media for health information [2]. In the United States, each year, pharmaceutical companies spend billions on direct-to-consumer (DTC) pharmaceutical drug advertising on various channels, with TV remaining the dominant medium by far [3]. DTC user-generated content on social media channels provides an interesting and important environment to study the conversations surrounding DTC drugs with high advertising budgets. DTC advertising, on any channel, has the potential to spark conversations among consumers. Those conversations on social media extend the word-of-mouth impact to a larger audience and provide a greater ability to monitor the conversation [4]. Recent publications express a continued need to better understand reactions and conversations about these drugs [5], given their large advertising budgets and the Food and Drug Administration (FDA)’s ongoing consideration of possible restrictions [6].

This study begins to illuminate the intersection between individuals’ desire to obtain health information online, strategic communication firms’ quest to promote clients’ brands through creativity and engagement, and the FDA’s mandate to oversee DTC drug advertising and protect public health. DTC drug conversations on social media have begun receiving more research attention, but one gap that remains is the use of social listening tools to better understand the content, sentiment, and trends surrounding DTC drugs on social media, beginning with those brands that spent the most from 2020 to 2022. This research contributes to scholarship in several ways, including agenda-setting research (what agendas might social media conversations set?). The managerial implications of what consumers are discussing online and how both advertising and public relations practitioners may need to address consumer concerns are also explored. Our research may help DTC advertisers understand the impact their creative output has, as expressed through consumers’ social media conversations, uses, and gratifications. Policymakers and pharmaceutical companies may also benefit from identifying the agenda-setting themes of social media conversations. This research aims to advance our understanding of where and how individuals are exposed to DTC drug information from a variety of sources in our fragmented and diversified media environment. We will first review some of what is already known in this area before describing our specific focus and social listening methods.

### DTC Advertising and User-Generated Content on Social Media

While TV still accounts for the vast majority of ad spending, the user-generated content and conversations surrounding DTC advertisements often occur online, and social listening facilitates our understanding of these massive datasets [7]. This study will help fill the gap in understanding what that online conversation consists of for the top spenders and will begin exploring the application of agenda-setting and uses and gratifications theories. As is often the case when an area is first being explored, the publications touch on a wide range of topics but do not include a broad content-based social listening study like ours. Papers that have been explored in this general area are summarized in Multimedia Appendix 1. After the inception of social media, research has investigated DTC on social media [8-11] to better understand broadly how pharmaceuticals were being promoted by their companies and how influencers may promote drugs [9], but little research has examined conversations and influencers used across drug categories. Several papers call for more research in this area [11,12].

Our study provides an important extension to this body of research by examining a broader sample of user-generated content, not just contents posted on company accounts or focused on only narrowly defined, albeit important, health concerns, such as obesity [13].

### FDA Regulations of Social Media DTC Ads

Because our study helps document the content of social media DTC drug conversations, we briefly review the guidance documents to provide the reader with a general understanding of the broader FDA regulatory and historical context. We summarize key points of the FDA guidance documents and urge interested readers to read the documents for key details related to agency expectations. Especially for readers who may not be familiar with DTC advertising regulation, this provides needed context for our study of Twitter (subsequently rebranded X) conversations of high-budget DTC drugs and offers guidance for future research. FDA regulation in this area is ever evolving, and the social media chapter is still being documented.

The FDA wrote several guidance documents in the 90s, including those related to broadcast and print DTC ads. The essence of those was that the ads must not be false or misleading, should present a fair balance of benefits and risks, should include the most important risk information, as well as the product indication and limitations, in consumer-friendly language, and should provide access to more detailed information often via a website [14]. The FDA also made provisions for “reminder” (also known as help-seeking) ads that did not need to include risk information when the benefits were not included alongside the brand name.

One of the most obvious additions to the FDA guidelines to address social media messaging [15] was the handling of the character space limitations present on Twitter, renamed X in July 2023, and other social media platforms. Twitter, prior to 2017, was limited to 140 characters, but that limit doubled in 2017. In 2023, Twitter began enabling subscribers to tweet up to 4000 characters. Throughout the remainder of this paper, we will use “Twitter” and “tweet” since the vast majority of our data were collected when the platform used those terms. The FDA acknowledges that the character limit is challenging, given the amount of information needed for DTC advertising, and does allow a reference, often a link, to access full risk information, though risk information must still be balanced, compared to benefits, and as prominently displayed [15].

Due to the fluid nature of online promotion, the FDA also released a draft guidance to help communication agencies handle “postmarketing” materials, such as dynamic, real-time promotional activities online, including blogs or social networking sites [16]. The FDA distinguishes between 3 main types of content—sites directly under the control of the firm (pharmaceutical company or their communication firm), third-party sites, and content generated on the firm’s behalf. In each of these instances, if the firm has even partial control (influence over content), then the firm is responsible for submitting to the FDA under the “postmarketing” submission requirements [16].

The third FDA draft guidance discusses third-party misinformation that may be posted online, and the FDA considers it voluntary whether the pharmaceutical company or its communication firm wants to correct the misinformation [17]. If the firm chooses to respond, the draft guidance provides more detail about how the response should be truthful and nonmisleading.

### Theoretical Frameworks

#### Overview

Two theoretical frameworks were utilized to guide our research, analysis, results, and future research suggestions. We chose these 2 theories to best understand the varied roles of individuals on social media: contributor to media content (agenda-setting) and consumer of content (uses and gratifications). Agenda-setting theory is useful for identifying which DTC drug topics are being discussed most frequently as these topics may be likely to impact engaged audiences, influence what they think about, and inform what industry, policymakers, and communicators may need to consider. Uses and gratifications theory has a natural fit with user-generated social content and provides a framework for understanding the content-related themes.

#### Agenda-Setting Theory

Agenda-setting theory has a history spanning over 50 years and encompasses multiple areas of mass communication research, including news media [18,19], social media [18], advertising [20], and health communication [21]. Agenda setting began as a way to describe how the news media establishes an agenda related to political campaigns and what the public considers important [22]. Decades of research have documented the strong relationship between the topics covered by the media and the issues people think are important [23]. Agenda setting provides a framework for understanding how social media may influence what individuals consider significant in relation to DTC drug advertising.

Although agenda-setting theory began with traditional news media, it has been applied to social media health promotion environments as well, specifically Twitter [4,21,24,25]. Yun et al [4] applied agenda setting to conversations on Twitter and examined key influencers to give recommendations for health campaigns based on their network analysis. This crucial area explores our understanding of which topics related to DTC drugs are being discussed and how they may influence individuals, the public, the media, organizations, and policy agendas.

#### Uses and Gratifications Theory

Uses and gratifications theory dates back to the 1940s, and the exploration aimed at better understanding why individuals choose certain media and what purpose it served them. Nearly 80 years and hundreds of publications later, Dolan et al [26] were among the first to suggest uses and gratifications theory’s applicability to social media settings. They identified 4 main groups of gratifying content for social media users based on a review of published studies: information, entertainment, remunerative (economic) benefits, and relational (social) benefits. These differ somewhat in important ways from the earlier typology that included diversion, personal relationships, personal identity, and surveillance (information) [27]. Uses and gratifications theory has been successfully applied to social media in a variety of studies, including associations with problematic internet use or addiction [28], impact on social relationships and psychological well-being [29], and Facebook usage [30]. However, little to no research has specifically applied uses and gratifications theory to conversation involving DTC-advertised drugs or the pharmaceutical industry.

Broadly speaking, individuals may obtain information about DTC drugs directly from advertisements, traditional media channels, or social media. This study aims to help fill a gap by identifying topics that may both provide individuals with uses and gratifications and set agenda items for the public to consider. The review of theory and literature led us to formulate two key research questions:

RQ1: What user-generated health-related topics discussed on social media mention DTC pharmaceutical drugs with the biggest DTC ad budgets? Which, if any, could be agenda-setting to the broader audience?

RQ2: What are the possible uses and gratifications that individuals engage in related to DTC drugs on Twitter? Additionally, are the mentions positive, negative, or neutral based on sentiment analysis?

## Methods

Our research used the social media analytics tool Brandwatch, which tracks online conversations across various social media channels. Social media “listening” has emerged as an important analytical method to investigate online social media content [31-33].

### Ethical Considerations

The Texas Christian University Institutional Review Board reviewed the research protocol and found the study to be exempt for human subjects research (2023-202). The authors adhered to personal, professional, and research ethics in conducting this research and preparing the manuscript, and they did not discern any conflict with the journal’s stated ethics. No informed consent or compensation was required as the review was exempt from human subjects review.

### Sampling

Given that very little research has applied social media listening techniques to DTC brands, we used purposive sampling and included the top DTC advertising spenders for 2020 to 2022, according to Kantar and Vivvix (listed in alphabetical order): Botox, Cibinqo, Dupixent, Eliquis, Entyvio, Humira, Jardiance, Nurtec ODT, Opdivo (+Yervoy), Otezla, Ozempic, Rexulti, Rinvoq, Rybelsus, Skyrizi, Tremfya, Trulicity, Verzenio, and Xeljanz [3,34-36]. Our rationale is that these brands may be most likely to be talked about on social media, given the preponderance of ads on traditional media. Two brands that spend significantly on digital formats, even with smaller overall budgets, were added to the sample to provide a broader, more diverse perspective—Orgovyx and Ponvory [3]. Multimedia Appendix 2 includes the study’s drugs, their primary condition treated, and their parent companies.

### Study Design and Query Construction

We collected historical and archived tweets from August 1, 2021, to August 1, 2023, related to a sample of top-spending DTC drug brands from 2020 to 2022 (Cibingo, Dupixent, Eliquis, Entyvio, Humira, Jardiance, Nurtec ODT, Yervoy, Otezla, Rexulti, Rinvoq, Rybelsus, Skyrizi, Tremfya, Trulicity, Verzenio, Xeljanz, Orgovyx, Ponvory, Botox, and Ozempic). We used these strategic keywords (drug brand names) as a query. We filtered the tweets to show only those in English and posted by Twitter accounts in the United States. Our first query gathered 486k total mentions and 247k unique authors. However, when analyzing the results, we found that most of these mentions came from only the keywords “Ozempic” and “Botox,” and these tweets focused on mentions not relevant to the drugs themselves or our research questions, such as popular culture or unrelated celebrity commentary, adversely affecting the remaining keywords’ results by masking the DTC pharmaceutical-oriented conversation. We removed Botox and Ozempic to facilitate a clearer analysis by eliminating noise caused by discussions that did not pertain to the research focus. It is important to acknowledge potential limitations in discerning the context of conversations related to these drugs, but the abundance of popular culture references unrelated to the study’s focus overshadowed the relevant mentions from other brands under examination and skewed the results. As a result, the keywords “Ozempic” and “Botox” were no longer included in the final query and sample.

After analyzing the query results, we found that spam tweets, deals and promotions, and automated bot tweets were also included in these mentions. Therefore, we refined the query and used Boolean operators (OR and AND NOT) to combine and exclude keywords and spam tweets that brought irrelevant results to the search (see Multimedia Appendix 3 for additional details on query construction) [37]. The new search results gathered a more appropriate number of mentions, 44.7k, for the second query during the selected time period. The sampling method involved capturing tweets over a specified period using relevant keywords to ensure a representative dataset. Sentiment analysis was conducted using Brandwatch’s natural language processing algorithms, which were further validated by a manual review to correct any discrepancies. Author analysis included examining tweet authors’ profiles to understand the contributors’ demographics and influence.

Brandwatch generated helpful data reports and organized them based on the search query topics into 3 main categories: measure, conversation, and people. The measure category included graphs that showcased the volume, content sources, top sites, sentiment, and emotion of all tweets. Additionally, Brandwatch generated a topics wheel, word cloud, and list of trending topics and top hashtags for the conversation category. Finally, the “People” section presented the general demographics of the Twitter users, such as gender, interests, profession, and influencers for the tweets in our data.

### Statistical Analysis

#### Topics Analysis

The topics wheel collected the most frequently mentioned topics in an interactive wheel graph. Each topic could be clicked to view a list of all the tweets containing that specific keyword. Similarly, the interactive word cloud displayed all the words, phrases, and entities most frequently found within the tweets. In a word cloud, the font size of a word is determined by how often the word is mentioned across tweets. The more frequently a word is used, the larger it appears in the word cloud. Like the topics wheel, clicking on each keyword showed the total number of mentions and the tweets for each mention. Each tweet included its publishing date, reach, and a link to the Twitter post and account profile.

We further analyzed the data using visualization methods (charts or other graphic tools) to discover patterns across mentions by applying thematic analysis techniques to the qualitative data captured through Brandwatch. Initially, Brandwatch’s artificial intelligence feature, Iris, generated preliminary categories. We then meticulously reviewed, reorganized, and refined these categories to ensure the accurate classification of sentiments and themes. This process followed the inductive, emergent approach recommended by Strauss and Corbin [38], involving open coding to identify initial themes and axial and selective coding to refine these themes into core categories. The topics for coding were selected based on their frequency and relevance to the discussion on Twitter about DTC pharmaceutical drugs. Relevant tweets included any mention that helped determine the type of content discussed on Twitter regarding DTC pharmaceutical drugs.

#### Custom Classifiers

We then used custom classifiers, Brandwatch’s machine-learning tool that uses the Brightview algorithm, to train custom classification models by adding mentions from our query results into different categories. The authors used a process similar to content analysis to agree upon how exemplar data should be classified. Once trained, the algorithm automatically assigns categories to each mention. The four main topics identified in the query for all other DTC drugs are (1) patient experiences, testimonials, or opinions; (2) high drug costs, insurance, or medical coverage; (3) pharmaceutical company or drug news; and (4) advertising discussions.

#### Sentiment Analysis

To determine attitudes, opinions, and emotions expressed by Twitter users, sentiment was analyzed using the Brandwatch sentiment score of the 50 most frequently used keywords, also shown in the word cloud (see Multimedia Appendix 4). Brandwatch determines the sentiment metric by weighing the number of positive and negative tweets for the keywords. A higher net sentiment indicates more positive than negative mentions, and a lower net sentiment indicates a higher ratio of negative to positive mentions.

Brandwatch identifies words and phrases that indicate positive or negative sentiment while setting rules that consider the effect context might have on the content’s tone. Because Brandwatch’s automated sentiment analysis process may miss the nuances of human communication (eg, sarcasm), we checked the positive, negative, and neutral mentions to verify that they were properly categorized.

Sentiment analysis is valuable for understanding public attitudes, opinions, and emotions on social media [39]. Analyzing DTC pharmaceutical advertising sentiment can provide crucial insights into how consumers perceive drug brands, their effectiveness, and associated risks. Research has shown that consumer emotions significantly influence decision-making, including health-related choices [40]. By examining sentiment in Twitter discussions, we can identify potential concerns, such as skepticism about a drug’s efficacy, fears of side effects, or distrust of pharmaceutical companies.

Brandwatch’s sentiment analysis uses advanced natural language processing techniques through machine learning and linguistic models to interpret social media conversations in context [40]. These models are designed to recognize slang, misspellings, and regional dialects, making them more effective at accurately classifying positive, negative, and neutral emotions. However, given the potential limitations of automated sentiment classification, manual verification is crucial for refining accuracy and reducing misclassification errors.

### Sample Description of Authors’ Posting

Understanding the demographic information of tweet authors is helpful for providing context to online conversations. Brandwatch determines the gender distribution of tweet authors by analyzing user profile details and post content. However, the exact methods used for gender inference remain proprietary and are not publicly disclosed.

The data provided by Brandwatch about the authors who posted the Twitter content help provide context about the content we described. Slightly more than half of the posts were categorized as men (Multimedia Appendix 5). The most common professions associated with these Twitter accounts included health practitioners, artists, executives, and teachers/lecturers. The authors’ top interests were family and parenting, politics, books, and beauty/health and fitness. The most mentioned influencers and authors included @BethWaldron (294 mentions), @pharmacychecker (243 mentions), @TheAsthmaCures (163 mentions), @newsfilterio (144 mentions), and @CrweWorld (102 mentions).

## Results

### Making Sense of the Data

We will first discuss the results of RQ1—what are the user-generated health topics that may be agenda setting—followed by RQ2—how might these topics suggest individuals’ uses and gratifications, including sentiment analysis. Between August 1, 2021, and August 1, 2023, there were a total of 44.7k mentions and 26.8k unique authors on Twitter for the search query related to the top-spending DTC drugs. Although mentions included earned, shared, and owned media about the topics of interest, the majority of mentions were shared and earned. The word cloud in Multimedia Appendix 4 included the top 50 most frequently used terms in the Twitter mentions for this time period. In addition to the size of the word, the shading of the word cloud indicates the volume of tweets associated with each keyword. The darkest shade of blue represents the words with the highest volume, while the lightest shade of blue represents those with the lowest volume.

We analyzed the most common words mentioned within the Twitter results through the word cloud derived from Brandwatch Insights. The top 10 most frequently used terms (Multimedia Appendix 6), contributing to a large majority of all mentions, are “drug” (5796 tweets), “month” (4276 tweets), “years” (3501 tweets), “cost” (3474 tweets), “dose” (3327 tweets), “COVID” (3092 tweets), “treatment” (2911 tweets), “eczema” (2845 tweets), “patients” (2845 tweets), and “vaccine” (2764 tweets). It is important to note that the number of mentions for the most frequently used words is not cumulative. For instance, if a single post includes the words “drug,” “month,” and “cost,” it will be counted as 3 separate mentions—1 for each keyword—despite originating from a single tweet.

Many keywords included mentions that covered more than one of the identified categories. Using Brandwatch’s OpenAI tool, Iris, and a close examination of the keywords in the word cloud, we found four main topics from the mentions gathered: (1) patient experiences, testimonials, or opinions; (2) high drug costs, insurance, or medical coverage; (3) pharmaceutical company or drug news; and (4) advertising discussions. Top keywords for each topic are listed in Textbox 1.

Textbox 1. **Keywords categorized by topics.**

1. Patient experiences, testimonials, or opinions

- side effects, years, patients, eczema got worse, blood, medication, taking, week, people

2. High drug costs, insurance, or medical cover

- COVID, dermatologist, month for dupixent, vaccine dose, told, years, paying, pocket, costs

3. Pharmaceutical company or drug news

- drug, cost, US, United States, cancer, dollars, prescription, biosimilar, treatment, lower

4. Advertising discussions

- commercial, song, ad, jingle, diabetes, woman, TV, people, Jardiance, commercials, big

### RQ1: Agenda-Setting Twitter DTC Drug Themes

#### Patient Experiences, Testimonials, or Opinions

The results from the keywords “years,” “patients,” “week,” “side effects,” “migraine,” “eczema got worse,” “told,” “life,” “blood,” “fed,” “medication,” and “control” also include a wide range of patient experiences and testimonials. Additionally, users discuss the side effects of various medications, such as Nurtec ODT, Humira, Jardiance, Rexulti, Xeljanz, Dupixent, Verzenio, and Eliquis. Concerns are expressed about the side effects of biologic medications, particularly Humira and Dupixent. Multiple users shared their experiences and side effects of taking drugs like Ozempic, detailing symptoms such as pale skin, lack of energy, constant body aches, and nausea. Notably, despite removing Ozempic from the sample due to its prevalence in popular culture references not relevant to the study, the term was still mentioned alongside other drug names. These mentions differed significantly as they directly reflected the experiences of patients, offering valuable insights into comparative analyses with other brands previously utilized by patients.

However, positive experiences with medications are also highlighted, with some tweets emphasizing the transformative effects of certain drugs, such as Dupixent improving the lives of individuals with eczema and Nurtec ODT providing relief for people experiencing migraine, overall improving their quality of life. For example, user @E_lisa_Beth tweeted, “I have had eczema since I was 2. I’ve had it just about everywhere. It got to the point my hands would split open. I couldn’t wear any type of sandals, b.c my feet were so bad. Been on dupixent for almost 4 years now. It is the miracle medication I’ve been waiting for my entire life.”

#### High Drug Costs, Insurance, or Medical Coverage

The keywords and phrases “cost,” “dermatologist,” “vaccine dose,” “pocket,” “month for Dupixent,” “years,” “paying,” “told,” and “COVID” were some of the most frequently mentioned words in conversations on Twitter that highlight a range of issues related to high drug prices, the cost of medications, and insurance dissatisfactions. Users compared drug prices, emphasizing the discrepancy in the cost of Humira between the United States and other countries, including Italy, Germany, and France. They also criticized pharmaceutical companies for what is perceived as price gouging, particularly in the case of top-selling drugs, such as Eliquis, Humira, Trulicity, and Revlimid, which, according to users, are significantly more expensive in the United States than in other countries (eg, @ChrisMurphyCT posted on Twitter “Cost of Humira: US: $3216 Italy: $526 Germany: $420 France: $248 When are we going to realize that our drug pricing policies just subsidize cheap drugs for the rest of the world? It’s a classic free rider problem.”).

A common topic to tweet about is the financial burden placed on patients because of high medication costs and the need for more affordable options. Furthermore, there is criticism of US drug pricing policies, with users suggesting that these policies result in subsidizing cheaper drugs in other countries. These comparisons highlight the substantial differences in drug pricing and raise questions about why drugs are more expensive in the United States compared to other nations. Some tweets mention the role of government, policymakers, and legislation in addressing the issue of high drug prices and negotiating prices with pharmaceutical companies to make medications more affordable for patients (eg, @Terryfor88 tweeted, “@amyklobuchar Could you look into the cost of Eliquis, a lifesaving necessary drug. It is very expensive even with Medicare and a supplement. There is no generic so drug companies need to lower the cost. Thank you.”).

Some individuals expressed their frustration with out-of-pocket expenses and the impact on their ability to afford necessary treatments, even with insurance coverage. Insurance and medication coverage concerns are also prevalent in Twitter conversations with the keywords “U.S.,” “United States,” “pocket,” and “insurance.” Users discussed issues with insurance coverage for medications like Eliquis and the need to switch to alternative drugs due to insurance policies. There were mentions of insurance companies denying coverage or requiring prior authorization for specific medications, leading to dissatisfaction among individuals regarding insurance plans and their handling of drugs like Entyvio, Humira, Mounjaro, Victoza, and Trulicity. For example, @LiliVicCreation tweeted, “I’m absolutely gutted about this Dupixent situation. My insurance is already denying my refill requests and I have no more medicine. I can’t believe these companies can just decide to stop covering medications. I hate private insurance companies.”

#### Pharmaceutical Company, Drug News, and Advertising Discussions

Because these 3 topics were somewhat intertwined, we present them in one combined section. Some Twitter discussions, including keywords such as “U.S.,” “United States,” “dollars,” “commercial,” “best-selling,” “biosimilar,” “Jardiance commercials,” and “treatment,” concerned pharmaceutical company practices and competition. Criticism was directed at pharmaceutical companies, particularly AbbVie, for increasing drug prices over time and delaying competition in the market. Users also expressed concerns about the implications of competition among drug companies, particularly blockbuster drugs such as Humira and Dupixent.

Moreover, some tweets expressed mixed feelings about the role of celebrity endorsements in pharmaceutical marketing, such as Lady Gaga endorsing Pfizer’s migraine drug Nurtec ODT, with some people asking Lady Gaga to “stop pushing pharmaceuticals on the public and her fans.” Additionally, criticism of the portrayal of individuals with overweight conditions was found in certain pharmaceutical commercials, leading to discussions about insulin costs and misconceptions about diabetes. For example, a user tweeted, "I’m sure they mean well but everyone in the new Jardiance commercial is obese. This fuels GOP insulin costs talking points. Type 1 diabetics are insulin dependent & it has nothing to do with weight. It runs in my family. My slim brother was dx with type 1 diabetes @ age 28.”

On the other hand, amusement and mockery of what users claimed to be “the cheesy and irritating” nature of pharmaceutical commercials made them “cringe.” Mentions revolve around the annoyance, particularly focusing on drugs like Jardiance’s and Skyrizi’s use of jingles and repetitive messaging. They also discussed how commercials often highlight countless side effects, pricing issues, and the overall saturation of drug advertisements in the media.

Users express mixed feelings about the Skyrizi ad, with some finding it amusing while others feel overwhelmed by its frequency. The commercial’s focus on various medical conditions and its musical approach has sparked discussions and reactions among viewers. A user wrote, “I still hate that Jardiance commercial. The little pill I hope goes straight to hell.” There are also discussions about the pharmaceutical industry’s advertising practices, as seen in commercials for drugs such as Jardiance or Skyrizi. The ads’ focus on specific medical conditions, side effects, and pricing issues has sparked debates about the effectiveness and ethics of DTC marketing in the health care sector.

The keywords and phrases “FDA,” “years,” “COVID,” “patients,” “effects,” “prescription,” “biosimilar,” “control,” and “cancer” are some that highlight various regulatory aspects of FDA approvals and drug treatment news, including those for different types of cancer and chronic diseases. Additionally, multiple tweets reported FDA updates regarding various medications, including label updates, approvals for treating specific conditions, diseases, or new indications, and warnings about compounded medicines and their use as substitutes for prescription drugs. Some tweets from news accounts announced the FDA’s approval of medications such as Rexulti and Dupixent for treating symptoms associated with dementia, specifically agitation and eosinophilic esophagitis, respectively. Other tweets announced the approval of drugs such as Rinvoq and Rexulti for treating particular diseases or conditions, such as Crohn disease, eosinophilic esophagitis, agitation in individuals with Alzheimer disease, and psoriatic arthritis (@LEAD_coalition tweeted, “FDA Approves First Drug to Treat Agitation Symptoms Associated with #Dementia due to #Alzheimer Disease https://www.fda.gov/news-events/press-announcements/fda-approves-first-drug-treat-agitation-symptoms-associated-dementia-due-alzheimers-disease by @US_FDA.”).

### RQ2: Topic Application to Uses and Gratifications

#### Sentiment Analysis

Examining how the topical themes indicate individuals’ uses and gratifications provides an additional layer of analysis and application. The 4 uses and gratifications we used came from the social media perspective, including information, entertainment, economic benefits, and social benefits [28], due to their applicability to the unique social media environment. We felt it was important to consider the sentiment analysis here to capture the positive or negative nature of these posts, as it helps capture the context of how the giver or receiver is feeling and the underlying tone of the posts.

**Figure 1.**
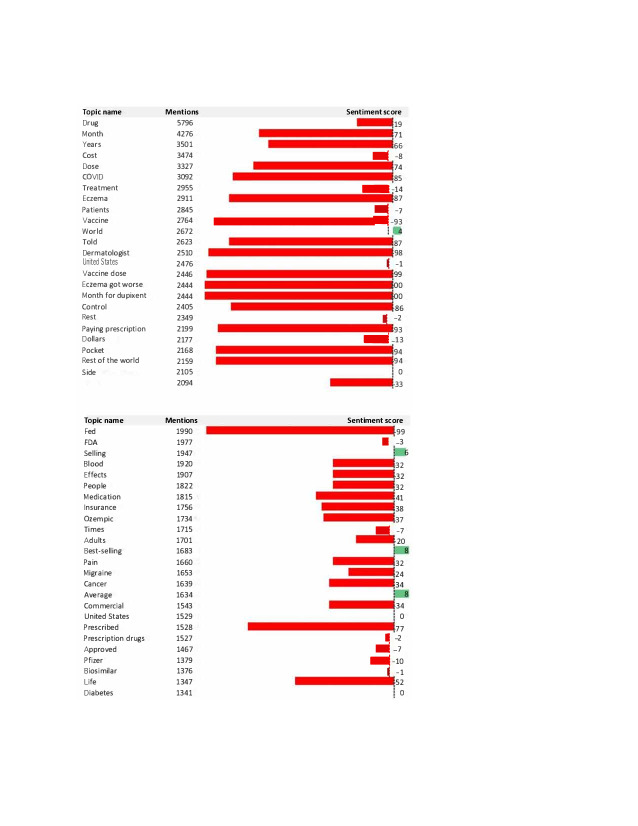
Query keyword mentions and sentiment score. FDA: Food and Drug Administration.

The overall net sentiment score for the top 50 keywords is negative, with 43 keywords having an average sentiment score of –47.7, 3 keywords with a score of 0, and 4 keywords with an average sentiment score of 6.5. Figure 1 shows each keyword’s total number of mentions and sentiment score.

#### Information

Some express a neutral sentiment, focusing on competition among pharmaceutical companies, including discussions about alternative drug options or biosimilars [41]. However, most neutral mentions encompass a wide range of earned media shared on Twitter, highlighting the achievements of pharmaceutical companies, drug advancements, or research, without indicating a favorable or unfavorable stance toward the subject matter. For instance, Fierce Pharma tweeted, “Boehringer Ingelheim posts more Jardiance growth as potential US kidney disease approval nears,” while linking their news article.

The effectiveness of various medications in treating conditions such as rheumatoid arthritis and colitis was another discussion reflecting some patients’ negative experiences. We listed these under information because individuals were often sharing their own experiences to help others who might benefit from knowing. Positive tweets mentioned users’ positive experiences and results with medications, such as Skyrizi, Humira, Rinvoq, and Trulicity. A user responded to a tweet that asked for suggestions for autoimmune illness-related eczema by describing how they were “grateful every day that I take Rinvoq for my autoimmune illness. Makes a world of difference. Five earlier meds were useless. Keep trying.” Similarly, another user replied, “I have been on Dupixent for eczema, and I also have RA so I very much feel you. Been on the Dupixent for just over a year, and it cleared 99% of my eczema which was mostly on my hands within a month or two. Absolute game changer for me and the only thing that’s ever worked.”

#### Entertainment

Tone was important for entertainment as some posts were sarcastic or made fun of the ads or creative elements within them. It might not be that the individuals found the ad entertaining but rather that they were attempting to entertain others with their commentary. Example tweets are included in Textbox 2.

Textbox 2. **Jardiance posts.**

The commercial for *Jardiance* with the lady dancing and singing is so stupid. How does one have a “touch” of diabetes?🤔

Ok I hate Pharma and I HATE Pharma advertising but real talk:

that *Jardiance* commercial kinda slaps 🫰🎶🎶🎶💃“I’m lowering myyyy alc!!! Here’s Hazel enjoying the commercial. User added a picture of their cat looking at the TV with the commercial in it.

#### Economic Benefits or Concerns

**Figure 2.**
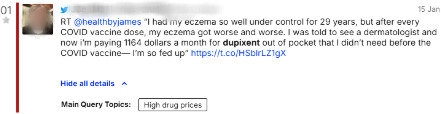
Dupixent screenshot.

**Figure 3.**
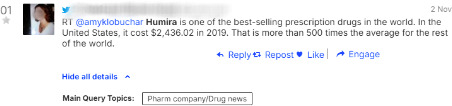
Humira screenshot.

Many of the negative mentions gathered in this analysis primarily focused on different aspects of health care and medication concerns, aligning with economic benefits—or in this case, concerns. Notable themes include reports of negative side effects following COVID vaccinations, particularly in the context of eczema worsening, which lead to costly prescriptions like Dupixent for symptom management (Figure 2). Medication prices and their impact on affordability were a prominent concern, with many individuals expressing frustration and apprehension about the high costs associated with drugs such as Humira, Eliquis, Jardiance, and Trulicity, sometimes resulting in discontinuation (ie, ceasing medication treatment) or financial hardships. Insurance coverage issues compounded the financial burden, particularly when obtaining specific drugs such as Humira and Entyvio. Figure 3 shows a post stating Humira is “one of the best-selling…” but costs most in the United States than the rest of the world.

Tweets also highlight users’ positive results from drug trials, such as Dupixent and Nurtec ODT, in treating chronic diseases, including chronic obstructive pulmonary disease, migraines, asthma, eczema, and more. These could honestly fit under information or social benefits, depending on the context of various cases.

#### Social Benefits

**Figure 4.**
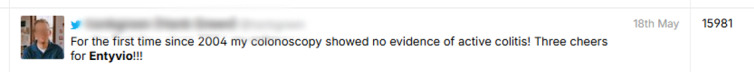
Entyvio screenshot.

Social benefits were often related to posts about how the drugs impacted their lives in a positive way or how grateful they were for the drug treatment, as shown in Figure 4.

While these were the only topics clearly tied to social and relational benefits, we know that there may be unspoken social benefits inherent in social media interactions; however, the tweets themselves did not display that content beyond these instances for our data and topic area.

## Discussion

Our study provides an important glimpse into what is being discussed on Twitter by, with, and about pharmaceutical drugs, which spend billions on advertising each year [3]. Although more tweets were neutral than positive or negative, the overall sentiment of the top terms was negative. The predominance of negative sentiment in our findings suggests that many social media users express apprehensions or criticisms about these drugs, highlighting the need for improved communication, transparency, and engagement strategies. These data are particularly valuable in pharmaceutical advertising, as they help identify consumer concerns, skepticism, and potential misinformation [38].

The content highlights a range of potentially agenda-setting topics, including pharmaceutical companies, their DTC drugs, patient experiences, costs, insurance, and advertising commentary. Twitter is serving as a forum for pharmaceutical companies, their constituents, and social media health influencers to discuss topics related to top-spending DTC drugs. To make the 44.7k mention number less arbitrary, our data indicate an average of 61 top-spending DTC drug mentions per day. We have certain hot topics and icons like Lady Gaga garner far more, but Twitter seems to be a popular place to connect with others about pharmaceutical concerns and thoughts.

We found both agenda-setting and uses and gratifications theories helpful for contextualizing our data. On Twitter, since anyone can post, the concept of agenda-setting broadens to include a variety of experts and nonexperts. The result is often a collection of opinions, perceptions, experiences, facts, and fictions. Key agendas emerge in the topical themes we identified regarding pharmaceutical companies, insurance, drug costs, and patient experiences. However, intent is not always clear; do people post with the intent of influencing a broader agenda? Some may want to share experiences for the cathartic nature of it, while others aim to influence the system or help others on their path. This need to connect has been noted in previous research [41] about online message boards, which were the precursors to social media.

This study demonstrates how Brandwatch can provide a high-level overview of the DTC drug conversation on social media. None of the topics are a surprise, but they empirically confirm what we may anecdotally believe is on the minds of consumers and patients. Professional DTC drug communicators need to be in tune with their audiences so that consumers do not perceive their messages as oblivious, out of touch, and only focused on their brand. We observe this disconnect between communication strategy (eg, singing and dancing) and patients’ headspace in the comments about “cheesy and irritating” nature of DTC ads that has also been noted in academic research [42,43]. DTC ads have a very challenging communication situation in balancing FDA guidance requirements, conveying technical medical terms in consumer-friendly ways, executing the branding strategy, satisfying client wishes, and connecting with an often-diverse audience. Tools such as Brandwatch and social media listening are key to staying relevant and up to date.

Uses and gratifications were not equally represented but were dominated by information, economic benefits, and entertainment. It was not evident how social benefits were sought or obtained. These findings are helpful for both academic researchers to understand the applications of this longstanding theory, as well as for policymakers to be aware of their constituents’ concerns. Some advertising practitioners, especially those with DTC drug accounts, may also benefit if they do not have access to tools such as Brandwatch. The data presented in this study help them to understand what topics are most often discussed for high-budget brands, as well as the uses and gratifications that social media users predominantly obtain.

This study also has implications for policymakers who oversee the health insurance, medical, and pharmaceutical industries. We found several themes related to regulation and pricing in these industries that policymakers and regulators would benefit from understanding better. DTC drug advertising is at this time only allowed in the United States and New Zealand and has garnered criticism since its start in the 1990s for increasing prices, among other things [44,45]. It is interesting that comments attributed high drug costs in the United States to subsidizing drugs in other countries as opposed to the cost of DTC contributing to what consumers pay.

Important next steps in this area of research may include a network analysis to examine the connections and interactions between various agents (eg, pharmaceutical companies, social media influencers, individuals, etc). This would reveal how the information flows through networks since our investigation focused solely on the content of the information. Another extension may be to include a wider variety of media budget beyond the top spenders among DTC drugs and see how volume and type of conversations may differ. A final extension may be to explore the qualitative and quantitative data to understand the why behind the what; since social media listening is similar to content analysis and cannot know why something was written. We encourage researchers to continue the application of agenda-setting and uses and gratification theories, especially in pursuit of cause-and-effect findings. Social media listening is most similar to content analysis and is not able to know what effect the content topics we documented are. Do the topics impact what consumers care about (agenda setting)? Do policymakers pursue legislation related to these key agendas? What is the motivation behind individuals’ uses and gratifications? Do these conversations impact consumer choice related to health conditions or DTC drug behaviors (eg, medical professional conversations, prescription adherence, or interpersonal word-of-mouth referrals)?

These ideas for future research would help to fill gaps left by the limitations of this study. Since this study focused on top-spending DTC drugs, it is important to look at a range of different spending levels and draw a probability sample to get a representative view of all DTC social media drug postings. It is also important to keep in mind that drug brands with a high advertising budget typically have also recently been approved for medical use by the FDA, act in a highly competitive market, and/or are under high public scrutiny. This means that a high advertising budget alone does not necessarily explain characteristics of the social media discourse on Twitter, and these other factors may also contribute to this study’s findings. Expanding the scope to include more platforms, beyond Twitter, would be another logical next step to compare differences and similarities. Furthermore, studies examining the content of messages are limited by not knowing what impact those messages have in terms of interaction, awareness, and understanding. These are key areas for future research. Future research may examine the connections between posts, topics, brands, and entity posting, perhaps using NodeXL or similar tools for conversation analysis. Qualitative investigations could explore the strategies or motivations of the individuals posting to delve even deeper.

A couple of other limitations the reader should consider include the sample description, historical events during the sample timeframe, and the difficulty in assessing sentiment. While estimates of tweet author descriptions promise useful insight into demographic trends, we must also recognize that this methodology may have inherent limitations. The categorization of keywords was not mutually exclusive, so the reader will notice that keywords could be shared between topic categories, resulting in an unavoidable overlap in naturally adjacent topics, such as patient experiences and drug costs.

## Supplementary material

10.2196/85641 Multimedia Appendix 1 Research involving direct-to-consumer advertising and user-generated content on social media.

10.2196/85641 Multimedia Appendix 2 Dataset of direct-to-consumer prescription drugs.

10.2196/85641 Multimedia Appendix 3 Details about the Brandwatch query.

10.2196/85641 Multimedia Appendix 4 Top 50 Twitter keywords in query data.

10.2196/85641 Multimedia Appendix 5 Description of authors.

10.2196/85641 Multimedia Appendix 6 Top 10 Twitter keywords for query data.

## Abbreviations

DTC: direct-to-consumer
FDA: Food and Drug Administration
