# Supplementary material for: Tweets Surrounding Pharmaceutical Drug Brands With Top Direct-to-Consumer TV-Advertising Budgets: Social Media Listening Study
Source: Online J Public Health Inform. 2026 Jun 18;18:e85641. doi: 10.2196/85641 (PMC13278610; doi:10.2196/85641)
Supplement: Multimedia Appendix 1 [file ojphi-v18-e85641-s001.docx]

Multimedia Appendix 1: Research involving DTC advertising and user-generated content on social media.

| **Study citation** | **Pharmaceutical company/DTC drug** | **Health condition** | **Social media channel/online content** | **Research method** | **Key findings** |
| --- | --- | --- | --- | --- | --- |
| Adams (2016) | 43 different including Humira, Cymbalta, and Vyvanse (most common) | 26 conditions, including asthma, plaque psoriasis, ADHD, and depression (most common) | Internet banner ads | Content analysis | - if banner ads were demonstrating fair balance and adequate provision - given “ambiguous regulatory guidelines” at the time “drug companies appear to make an attempt to adapt regulatory guidelines designed for traditional media” - recommended FDA formalize banner ad requirements |
| Chesnes and Jin (2016) | 373 different drugs, including top 10 spenders: Lipitor, Cymbalta, Cialis, Advair, Abilify, Symbicort, Lyrica Pregabalin, Plavix, Viagra, and Pristiq | Various including cardiovascular health, mental health, sexual health, asthma, and fibromyalgia | Online search | Descriptive and regression analysis of secondary statistical data | - a direct relationship between DTC spending and online search indicating that DTC ads increase searches for the advertised drug and other drugs and remedies in that category |
| Curtis (2017) | Various arthritis medications including Xeljanz | arthritis | Twitter, Facebook, blogs, and discussion boards | Social media data analysis using Treato platform | - how DTC drug advertising’s impact on social media posts for arthritis drugs and the finding that safety concerns were more common than favorable posts |
| Fogel (2019) | n/a | various | DTC ads on YouTube, Facebook, Twitter, and various online sites | survey | - College students go to social media content for DTC over online or traditional media; Twitter was most associated with obtaining a prescription drug without a prescription |
| Fogel (2023) | n/a | various | various | survey | - how social factors, like subjective norms and trust, impacted college students’ intentions after seeing DTC on social media |
| **Study citation** | **Pharmaceutical company/DTC drug** | **Health condition** | **Social media channel/online content** | **Research method** | **Key findings** |
| Haggerty (2022) | No brands just terms like “diet pills” | obesity | Twitter | NCapture to collect Tweets and NVivio for qualitative analysis | - how people on Twitter communicate about obesity and related stigmas, including personal blame and dangers associated with taking weight loss medications |
| Hugmann (2016) | Top 50 global pharmaceutical manufacturers, including Johnson & Johnson, GlaxoSmithKline, Novo Nordisk, Pfizer, Novartis, Boehringer Ingelheim, Bayer Healthcare, Merck & Co., Astra Zeneca, and UCB | n/a | Facebook, Twitter, YouTube, and LinkedIn | content analysis | - how pharmaceutical manufacturers were using social media and if their posts followed FDA guidance documents |
| Jain (2020) | None listed, only looked at opioids as a class of drugs | opioid use and abuse | Twitter | content analysis | - what content is shared on Twitter about opioids - use of the word “addiction” made retweeting less likely |
| Kim (2015) | Not reported | 21 categories with highest warning letters being cancer, dermatology, urology, and cardiovascular | 95% of alleged violations were on branded drug websites, online paid ads, and in online videos | content analysis | - analysis of DTC online promotion-related warning letters that found that majority of alleged violations involved lack of risk information and misrepresentation of efficacy - suggested this indicates the issue of fair balance, risk versus benefit information, was a key concern based on the analysis |
| articles commenting on Kim 2015 (Carpentier, 2016; Mackey, 2016; Southwell, 2016) | n/a | n/a | n/a | n/a | - discussing the new DTC format at the time in advance of most of the more recent FDA guidance documents |

| **Study citation** | **Pharmaceutical company/DTC drug** | **Health condition** | **Social media channel/online content** | **Research method** | **Key findings** |
| --- | --- | --- | --- | --- | --- |
| Tyrawski (2015) | top 15 international pharmaceutical companies | accounts for the top 20 2013 U.S. drugs | Facebook, Twitter, and YouTube | content analysis | - all but one of the drugs had social media accounts, with Twitter being the most common (90%), followed by Facebook and YouTube (both at 66%) - These posts, most being help-seeking without product claims, also examined user-generated content/comments on the pages |
| Willis (2022) | n/a | n/a | n/a | none, literature review and research agenda | - literature review related to online patient “influencers” being used to promote DTC drugs and how they may help pharmaceutical companies improve relationships with their consumer |
| Willis (2023) | n/a | various, including diabetes, celiac disease, IBS, chronic migraine, cancer, asthma, and fibromyalgia | n/a | in-depth interviews | - how social media health influencers communicate health literacy for pharmaceuticals - used the Health Belief Model as theoretical guide - three themes were “understanding disease through experience,” staying informed on the science or field,” and “suggesting that physicians know best.” |
| Wombacher (2020) | n/a | drug use including illegal substances | Reddit, r/Drugs | content analysis | - what types of social support were given on reddit for various drugs, including illegal substances - primary topics included drug effects and drug dosages |
