# Supplementary material for: Tweets Surrounding Pharmaceutical Drug Brands With Top Direct-to-Consumer TV-Advertising Budgets: Social Media Listening Study
Source: Online J Public Health Inform. 2026 Jun 18;18:e85641. doi: 10.2196/85641 (PMC13278610; doi:10.2196/85641)
Supplement: Multimedia Appendix 2 [file ojphi-v18-e85641-s002.docx]

Multimedia Appendix 2: Dataset DTC prescription drugs.

| **DTC Prescription Drug** | **Primary Treatment** | **Parent Company** |
| --- | --- | --- |
| ~~Botox^[[1]](#footnote-1)^*~~ | ~~Dermatological, wrinkle treatment~~ | ~~Allergan, AbbVie~~ |
| Cibinqo | eczema | Pfizer |
| Dupixent | eczema | Regeneron and Sanofi |
| Eliquis | Serious blood clot prevention | Pfizer and Bristol-Myers Squibb |
| Entyvio | ulcerative colitis, Crohn's disease | Takeda |
| Humira | rheumatoid arthritis | AbbVie |
| Jardiance | type 2 diabetes | Eli Lilly and Boehringer Ingelheim |
| Nurtec ODT | migraines | Pfizer |
| Opdivo (+Yervoy) | cancer | Bristol-Myers Squibb |
| Orgovyx | cancer | Pfizer and Sumitoma Pharma |
| Otezla | psoriasis and psoriatic arthritis | Amgen |
| ~~Ozempic^[[2]](#footnote-2)^*~~ | ~~type 2 diabetes~~ | ~~Novo Nordisk~~ |
| Ponvory | multiple sclerosis | Janssen |
| Rexulti | major depressive disorder | Otsuka and Lunbeck |
| Rinvoq | rheumatoid arthritis | AbbVie |
| Rybelsus | type 2 diabetes | Novo Nordisk |
| Skyrizi | plaque psoriasis | AbbVie |
| Tremfya | plaque psoriasis | Johnson & Johnson |
| Trulicity | type 2 diabetes | Eli Lilly |
| Verzenio | cancer | Eli Lilly |
| Xeljanz | rheumatoid arthritis | Pfizer |

1. * Botox and Ozempic removed due to excessive popular culture references not related to study focus, facilitating a more accurate analysis. [↑](#footnote-ref-1)
2. [↑](#footnote-ref-2)
