# Supplementary material for: Tweets Surrounding Pharmaceutical Drug Brands With Top Direct-to-Consumer TV-Advertising Budgets: Social Media Listening Study
Source: Online J Public Health Inform. 2026 Jun 18;18:e85641. doi: 10.2196/85641 (PMC13278610; doi:10.2196/85641)
Supplement: Multimedia Appendix 3 [file ojphi-v18-e85641-s003.docx]

Multimedia appendix 3: Details about Brandwatch query.

Boolean operator OR was used to broaden the search to include results that contain either or all keywords, the name of the DTC drugs, within the query. The operator NOT was also used to exclude unwanted terms found, within spam tweets or unrelated tweets to the topics, such as the keywords "email," "billion," "Q2," "$LLY," and "Cow."

(ozempic OR botox) AND NOT (appointment OR schedule OR book OR contact OR "visit us" OR "call us" OR explore OR "Republican National Committee" OR RNC OR republican OR democrat* OR AOC OR Trump OR "African Korean" OR MAGA OR Carlson OR Pelosi OR whitening OR election OR left OR right OR #trumpmugshot OR #royalrumble OR "elon musk")

((cibingo OR dupixent OR eliquis OR entyvio OR humira OR jardiance OR "nurtec ODT" OR yervoy OR otezla OR rexulti OR rinvoq OR rybelsus OR skyrizi OR tremfya OR trulicity OR verzenio OR xeljanz OR orgovyx OR ponvory) AND NOT (email OR billion OR Q2 OR $LLY OR Cow))
