# Supplementary figures and images for: Tweets Surrounding Pharmaceutical Drug Brands With Top Direct-to-Consumer TV-Advertising Budgets: Social Media Listening Study
Source: Online J Public Health Inform. 2026 Jun 18;18:e85641. doi: 10.2196/85641 (PMC13278610; doi:10.2196/85641)

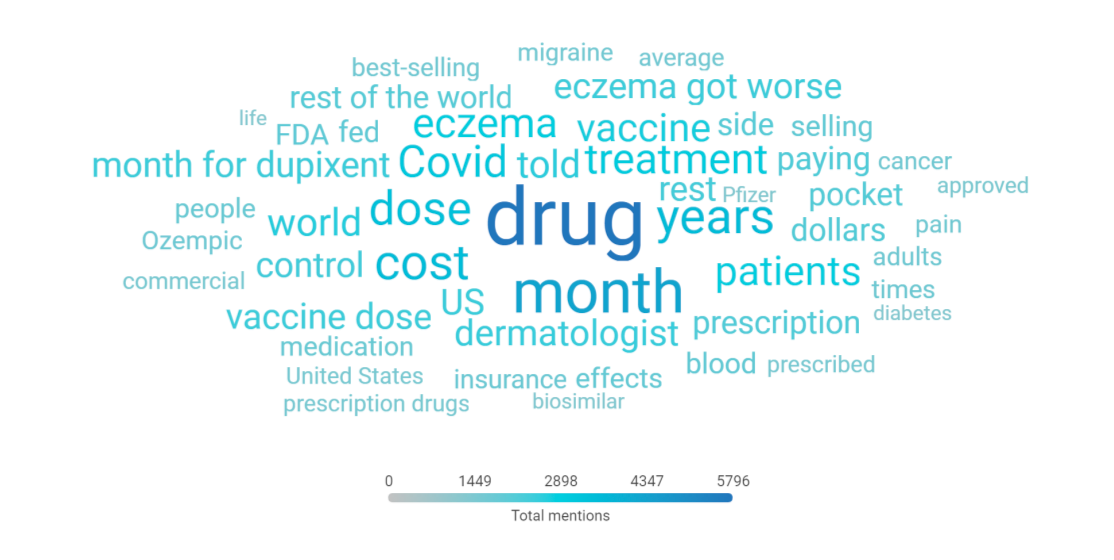

Supplement: Multimedia Appendix 4 [file ojphi-v18-e85641-s004.png]

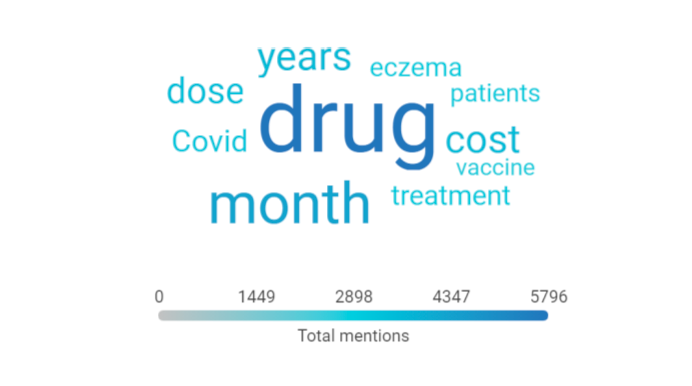

Supplement: Multimedia Appendix 6 [file ojphi-v18-e85641-s006.png]
