# Supplementary material for: Tweets Surrounding Pharmaceutical Drug Brands With Top Direct-to-Consumer TV-Advertising Budgets: Social Media Listening Study
Source: Online J Public Health Inform. 2026 Jun 18;18:e85641. doi: 10.2196/85641 (PMC13278610; doi:10.2196/85641)
Supplement: Multimedia Appendix 5 [file ojphi-v18-e85641-s005.pdf]

Gender split

45% — 5 005 authors

55% — 6 131 authors

TOP INTERESTS	UNIQUE AUTHORS	
Family & Parenting	3 396	16%
Politics	2 456	12%
Books	1 837	9%
Beauty/Health & Fitness	1 742	8%

TOP PROFESSIONS	UNIQUE AUTHORS	
Health practitioner	1 040	19%
Artist	933	17%
Executive	814	15%
Teacher & Lecturer	705	13%
